# Supplementary material for: A Polyaminobiaryl-Based β-secretase Modulator Alleviates Cognitive Impairments, Amyloid Load, Astrogliosis, and Neuroinflammation in APPSwe/PSEN1ΔE9 Mice Model of Amyloid Pathology
Source: Int J Mol Sci. 2023 Mar 9;24(6):5285. doi: 10.3390/ijms24065285 (PMC10048993; doi:10.3390/ijms24065285)
Supplement: Supplementary file 1 [file ijms-24-05285-s001.zip › ijms-2159929-supplementary.pdf]

SUPPLEMENTARY FIGURE S1

A

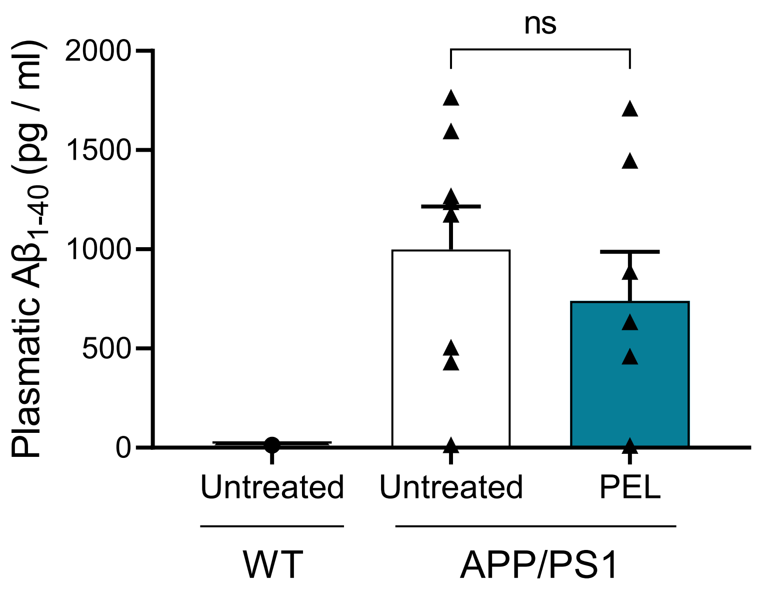


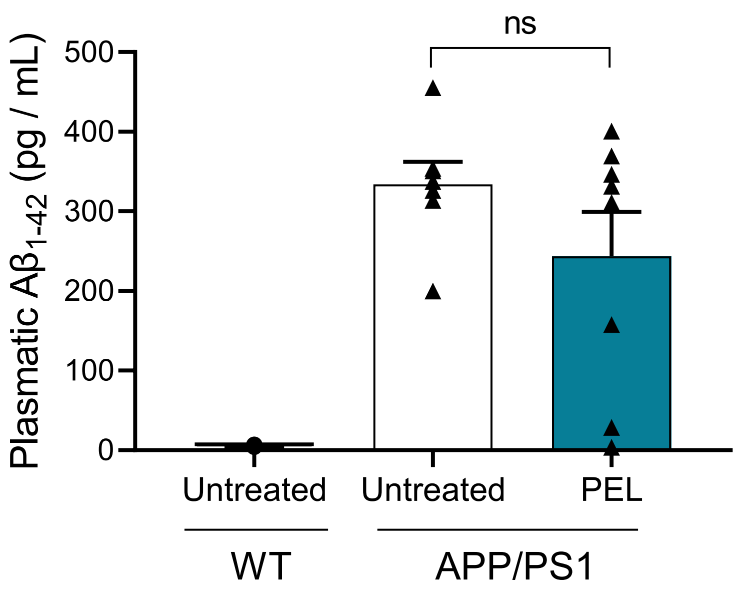

SUPPLEMENTARY TABLE S1

| Name | Species | Dilution | Buffer | Reference |
|---|---|---|---|---|
| Western Blot – Detection antibodies | | | | |
| **GAPDH** | Rabbit | 1 : 50 000 | TNT/5% milk | Sigma G9545 |
| **PP2A$_C$ subunit** | Rabbit | 1 : 1000 | TNT/5% milk | Sigma 07-324 |
| **APP-C17** | Rabbit | 1 : 7500 | TNT/5% milk | Homemade 100354 2071 |
| **PhosphoAPP (pThr668)** | Rabbit | 1 : 2000 | TNT/5% milk | Homemade |
| **GFAP** | Rabbit | 1 :10000 | TNT/5% milk | Sigma G9269 |
| **6E10** | Mouse | 1 : 1000 | TNT/5% milk | Biolegend 803001 |
| **CDK5 (J3)** | Mouse | 1: 500 | TNT/5% BSA | SantaCruz sc6247 |
| **GGA1 (3F11)** | Mouse | 1: 500 | TNT/5% BSA | Sigma-Aldrich WH0026088M1 |
| **GSK3-β (Y216)** | Rabbit | 1 : 1000 | TNT/5% BSA | SantaCruz sc135653 |
| **Fyn (H-80)** | Rabbit | 1 : 1000 | TNT/5% milk | SantaCruz sc2879 |
| Western Blot – Revelation antibodies | | | | |
| **Goat anti-Rabbit** | Goat | 1 : 5000 | | Vector PI1000 |
| **Goat anti-Mouse** | Goat | 1 : 50 000 | | Merck Millipore AP200P |
| Immunohistochemistry – Primary antibodies | | | | |
| **Biotin anti β-amyloid 17-24 (4G8)** | Mouse | 1 : 1000 | | Biolegend SIG-39240 |
| **GFAP** | Rabbit | 1 : 1000 | | Dako, Z0334 |
| Immunohistochemistry – Secondary antibodies | | | | |
| **Alexa Fluor 568 goat anti-Rabbit** | Goat | 1 : 1000 | | Invitrogen A11011 |
| **Anti-rabbit biotinylated** | Goat | 1 : 400 | | Vector BA-1000 |

SUPPLEMENTARY TABLE S2

| Primers | Accession number | Forward primer | Reverse primer | Amplicon length |
|---|---|---|---|---|
| CCL3 | NM_011337.2 | 5'-TGCCCTTGCTGTTCTTCTCT-3' | 5'-GTGGAATCTTCCGGCTGTAG-3' | 112 |
| CCL4 | NM_013652.2 | 5'-GCCCTCTCTCTCCTCTTGCT-3' | 5'-GAGGGTCAGAGCCCATTG-3' | 72 |
| CLEC7A | NM_020008.2 | 5'-ATGGTTCTGGGAGGATGGAT-3' | 5'-GCTTTCCTGGGGAGCTGTAT-3' | 72 |
| CONNEXIN43 | NM_010288.3 | 5'-GTGCCGGCTTCACTTTCA-3' | 5'-GGAGTAGGCTTGGACCTTGTC-3' | 97 |
| C1QA | NM_007572.2 | 5'-GGAGCATCCAGTTTGATCG-3' | 5'-CATCCCTGAGAGGTCTCCAT-3' | 60 |
| C3 | NM_009778.3 | 5'-CAGCCTTCGTCCTCATCG-3' | 5'-ATGCTCCCAGGAAGGCTATT-3' | 76 |
| GFAP | NM_001131020.1 | 5'-CGCGAACAGGAAGAGCGCCA -3' | 5'-GTGGCGGGCCATCTCCTCCT-3' | 104 |
| ITGAX | NM_021334.2 | 5'-ATGGAGCCTCAAGACAGGAC-3' | 5'-GGATCTGGGATGCTGAAATC-3' | 62 |
| LCN2 | NM_008491.1 | 5'-CCATCTATGAGCTACAAGAGAACAAT-3' | 5'-TCTGATCCAGTAGCGACAGC-3' | 89 |
| TLR2 | NM_011905.3 | 5'-GGGGCTTCACTTCTCTGCTT-3' | 5'-AGCATCCTCTGCGATTTGACG-3' | 110 |
| CYCLOPHILIN A | NM_008907.1 | 5'-AGCATACAGGTCCTGGCATC-3' | 5'-TTCACCTTCCCAAAGACCAC-3' | 126 |
